# Preclinical Trial of *Ocotea puberula* (Rich.) Nees (“Canela-Guaicá”) in Wound Healing: Validation of a Traditional Medicine Practice Used by Indigenous Groups in Southern Brazil

**DOI:** 10.1155/2023/3641383

**Published:** 2023-02-09

**Authors:** Guilherme Arcaro, Adriana Yuriko Koga, Bruna Carletto, Giovana Manfron Budel, Maria Dagmar da Rocha Gaspar, Jessica Mendes Nadal, Andressa Novatski, Leandro Cavalcante Lipinski, Paulo Vitor Farago, Luís Antonio Pinheiro

**Affiliations:** ^1^Postgraduate Program in Health Sciences, State University of Ponta Grossa, Ponta Grossa, Brazil; ^2^Postgraduate Program in Pharmaceutical Sciences, State University of Ponta Grossa, Ponta Grossa, Brazil; ^3^Santo Anjo High School, Curitiba, Brazil

## Abstract

**Background:**

“Canela-guaicá,” “guaicá,” or “canela-sebo” [*Ocotea puberula* (Rich.) Nees] is a native species that is traditionally used by Kaingang indigenous groups for wound healing in southern Brazil. The aim of this study was to extract the mucilage from *O. puberula* barks, perform its phytochemical and physicochemical characterization, and investigate its healing potential.

**Methods:**

A murine wound model was used as a preclinical trial for authentication of the traditional knowledge from Kaingang indigenous communities.

**Results:**

Alkaloids and polysaccharides were identified by usual qualitative reactions and Fourier-transform infrared spectroscopy. This natural product showed thermal stability and pseudoplastic properties that were considered suitable for the intended use. A higher initial exacerbation of inflammatory response after 7 days, an improved angiogenesis after 14 days, and an increased wound shrinkage after 21 days were statistically significant for the “canela-guaicá” bark extract in the preclinical trial when compared to the silver calcium alginate dressing (positive control).

**Conclusion:**

The healing potential of the “canela-guaicá” bark extract, traditionally used by the Kaingang indigenous community from southern Brazil, was preclinically validated. This study paves the way for designing novel wound dressings containing this natural product in order to treat acute and chronic wounds.

## 1. Introduction

Chronic wounds are a major health problem with high costs and impacts on care programs, primarily affecting adults and elderly individuals. In the UK's National Health Service (NHS), the cost of wound care is conservatively estimated at $54 billion, including various outpatient facilities. In developed countries, it has been estimated that 1–2% of the whole population may suffer from chronic wounds at some point in their lifetime [1–3].

Wound healing is a complex process that involves an orderly sequence of events, divided into four steps: hemostasis, inflammation, proliferative, and remodeling phase [4]. There are commercial dressings for treating acute and chronic wounds that include silver, nanocrystalline silver, alginate, and hydrogel [5–7].

However, this dressing might not be completely effective due to their high cost and prolonged time for treating chronic wounds, such as ulcers and scars, which remain as unresolved health problems [1]. In that sense, innovative polymer membrane dressings based on natural products can be a suitable alternative to improve the wound healing process [8–11].

*Ocotea puberula* (Rich.) Nees is an evergreen tree that belongs to the Lauraceae. Its barks present the well-known aporphine alkaloids as dicentrine, which demonstrated antituberculosis activity and antinociceptive effects [12, 13]. In addition, there are studies that the mucilaginous gel can be used for wound healing, as reported by taxonomic and ethnopharmacological studies [6, 14]. However, no previous report was devoted to investigate the wound healing effect of the mucilaginous “canela-guaicá” bark extract (CGBE) in order to provide an ethnopharmacological-based strategy for wound repair.

The aim of this paper was to characterize the mucilaginous CGBE and to treat standardized lesions in a wound healing preclinical model by comparing it with a commercially available ionic silver wound dressing.

## 2. Methods

### 2.1. Plant Material and Processing

Samples of barks of *Ocotea puberula* (Rich.) Ness, Lauraceae, were collected from six tree specimens at the State University of Ponta Grossa, Ponta Grossa, Parana, Brazil (latitude 25°05′39″S and longitude 50°06′05″W) in October 2018. The plant material was identified, and a voucher specimen was stored under the number HUPG 19107 at the Herbarium of the State University of Ponta Grossa. *Ocotea puberula* barks were cut into 4 cm^2^-sized fragments, dried at 25 ± 2°C, and then ground into a Wiley-type knife mill (Solab, SL31 model, Piracicaba, Brazil) to obtain a 500 *μ*m sized powder.

This standardized powder was stored in sealed containers in dark conditions at room temperature (20 ± 2°C).

### 2.2. Preparation and Characterization of “Canela-Guaicá” Bark Extract

The “canela-guaicá” bark extract (CGBE) was prepared in triplicate using 20 g of the previously standardized powder of *Ocotea puberula* barks and 800 mL of freshly obtained distilled water (Milli-Q EQ 7000 ultrapure water system, Millipore, Burlington, MA, USA) by stirring (Fisaton stirrer, 713 model, São Paulo, Brazil) at 60 ± 2°C and 300 rpm for 1 h. The extract was filtered through cotton wool to remove solid particles and then centrifuged twice at 20 ± 2°C and 3600 × g for 15 min. Finally, ultracentrifugation at 8 ± 1°C and 15000 × g for 10 min was carried out in order to obtain a soluble fraction. The mucilaginous CGBE was obtained after precipitation at 4 ± 2°C for 24 h using ethanol 95% (Vetec Quimica Fina, Duque de Caxias, Brazil) at 1 : 3 volume ratio (soluble fraction/ethyl alcohol).

Preliminary qualitative assays were carried out in order to confirm the nature of the CGBE extract obtained. The chemical tests included were: Molish's test for carbohydrates, ruthenium red test for mucilage, Benedict's test and Fehling's test for reducing/nonreducing sugar, ninhydrin test for proteins, Folin–Ciocalteu test for total phenolic content, Shinoda test for flavonoid, ferric chloride test for tannins, and Dragendorff's test for alkaloids [15–17].

The freeze-dried CGBE was investigated by thermogravimetric analysis (TGA) and derivative thermogravimetric analysis (DTG). TGA and DTG analyses were both performed using the STA 6000 instrument (Perkin Elmer, Waltham, MA, USA) with a mass of 5 mg CGBE into an alumina crucible. The equipment was previously calibrated using indium (In, M.P. = 156.6°C, Δ*H* fusion = 28.54 J/g) and was heated at a 10°C/min constant rate, from 20 to 600°C, under constant nitrogen flow (50 mL/min) [18].

The CGBE rheological profile was performed in triplicate using a Discovery DR2TA-Instrumentals hybrid rheometer (New Castle, DE, USA) coupled to the TC81 Peltier temperature controller kept at 36.5°C (similar to human skin temperature) and to the PP40Ti plate-plate sensors. A volume of 20 mL of the mucilaginous CGBE was placed on the Peltier plate in order to obtain the complex viscosity data including storage (*G*′) and loss (*G*″) after moduli. The rheological analysis was carried out using 30 s immersion time, 0.5 Pa tension, and logarithmic weep with 0.1 to 100 rad/s angular frequency and 5 points per decade.

The fourier-transform infrared (FTIR) spectrum was recorded by using a KBr tablet (4 mg of CGBE and 196 mg of spectroscopic grade KBr, 2% w/w) in the 4000–400 cm^–1^ range, with a resolution of 4 cm^–1^ at 32 scan/min in the IR Prestige-21 spectrophotometer (Shimadzu, Kyoto, Japan) [19].

### 2.3. Preclinical Trial: Wound Healing Assay

All the experiments were approved by the Ethics Committee on Animals Experimentation of the State University of Ponta Grossa (CEUA/UEPG) under process number 016/2018. The wound healing assay was carried out in accordance with the ARRIVE Guidelines [20]. The sample number was calculated using the Gpower 3.1 program (2020 version, Heinrich Heine University Düsseldorf, Düsseldorf, Germany) at 80% test power based on previous studies [4, 21].

The animals were selected in accordance with age and weight (standardized age approximately 3 months, weighing around 290 g to 380 g). The rats were weighed and separated by the probabilistic random-choice process into 3 groups of 15 animals each, amounting to a 45 Male Wistar *Rattus norvegicus albinus*. These animals were kept in the light-dark cycle of 12 hours, temperature of 21 ± 2°C, adequate ventilation system, and free access to food and water.

For the surgical procedure, rats were intraperitoneally (i.p.) anesthetized with 40 mg/kg of 10% ketamine and 10 mg/kg of 2% xylazine [22]. The back of each animal was shaved, and a dorsal lesion (2 cm × 2 cm) was made with a scalpel up to the level of the aponeurotic tissue, and then the lesions were cleaned with 0.9% sterile saline solution. Each treatment was then applied every other day using an aseptic procedure. The animals were divided into 3 groups: positive control (PC)−4 × 4 cm foam of alginate containing silver (Megisorb Ag, Mölnlycke Health Care, Mölnlycke, Sweden); negative control (NC)−5 mL of 0.9% sterile saline solution (Fresenius Kabi, Aquiraz, Brazil), and CGBE (5 g of mucilaginous gel). CGBE presented a mean dry matter content of 105.2 mg/g and showed mucilage (92.1 mg/g) and alkaloids (43.6 *μ*g/g) as the most representative chemical biomarkers. All lesions were covered with sterile bandages.

Each wound was macroscopically inspected after 7, 14, and 21 days and photographed by standardized apparatus. The wound healing index (WHI) was obtained as a percentage of re-epithelialization using the lesion areas provided by standard photographs assessed by Image J 1.44 software (National Institute of Health, Bethesda, MD, USA) on days 0, 7, 14, and 21. The initial areas represented the lesion areas on the day of the surgical procedure, and the final areas corresponded to the lesion areas on each day of euthanasia. Then, the WHI value was calculated by the following formula WHI (%) = [(initial area − final area/initial area)]*∗*100.

At each time interval, 5 animals were euthanized per group for histochemical biopsies. Skin fragments were picked up through a skin incision, coating the healthy skin and the preinjured area. A part of the material was promptly fixed in 10% phosphate-buffered formalin (pH 7.3 and 0.1 M) for 24 hours. Subsequently, the specimens were dehydrated in ethyl alcohol in ascending concentrations, diaphanized by xylol, and impregnated with liquid paraffin in an oven set at 59°C and embedded in paraffin. The blocks were cut on a Minot microtome (LEICA®, model M2125RT), adjusted to five micrometers, and processed for hematoxylin (Merck, Darmstadt, Germany) and eosin (Merck, Darmstadt, Germany) (HE) staining to evaluate inflammatory cells and angiogenesis. Masson's trichrome staining (Biognost, Zagreb, Croatia) was used to evaluate collagen fibers. The analyses were accomplished using a light microscope (LEICA® DM500 Trinocular Microscope, Wetzlar, Germany) coupled with an ICC50 HD camera, and the images were digitalized by computer using LEICA® LAS EZ software (Wetzlar, Germany).

All analyses were performed blindly by two evaluators since the histological slides were identified by coding to which the examiner had no knowledge of which group of animals these slides belonged to. In the morphohistological evaluation, inflammatory infiltrate, vascular formation, neocollagenesis and re-epithelialization were observed at all times, according to the standard protocol for histological evaluation [20]. The scores attributed to these histological analyses were absence (0), presence (1), intensely infiltrated (2), and severely infiltrated (3) [4, 21].

The statistical data were obtained using the GraphPad Prism 5 program (San Diego, CA, USA). The results were expressed as mean ± standard deviation. The one-way ANOVA and Tukey's post hoc test were used for the histological assays. Values were considered significant when *p* < 0.05.

## 3. Results

### 3.1. Preparation and Characterization of “Canela-Guaicá” Bark Extract

The dried and powdered barks of *O. puberula* showed a suitable yield of freeze-dried mucilage (13.43%). The phytochemical results demonstrated the remarkable presence of carbohydrates and mucilage in CGBE using Molish's test and ruthenium red test, respectively. Benedict's test and Fehling's test revealed no change in color, which corresponded to the presence of nonreducing sugar. The positive result was also observed using Dragendorff's reagent, which means that CGBE contains alkaloids. Other photochemical assays for proteins, phenolic compounds, flavonoids, and tannins presented negative results.

The presence of phytochemicals in *O. puberula* barks mainly represented by mucilage corroborates with the indigenous use of CGBE for wound healing. The water retention capacity along with the moisturizing properties makes mucilages ideal for the production of dressings for wounds and skin burns [23, 24]. In particular, mucilage from CGBE can also act on healing due to the fact that it is biocompatible and nontoxic since it is composed of carbohydrates. Furthermore, the CGBE is a herbal traditional practice provided by the culture-nature relations of Kaingang groups in southern Brazil and is an ethnobotanical approach for valuing the biodiversity of the Brazilian flora at the same time. There is an extensive report about the presence of aporphine alkaloids in *O. puberula* with antinociceptive effects by nullifying or reducing the perception and transmission of pain-causing stimuli [12, 13]. These alkaloids can increase the wound contraction rate and can reduce the time interval for epithelialization [25, 26].

**Figure 1:**
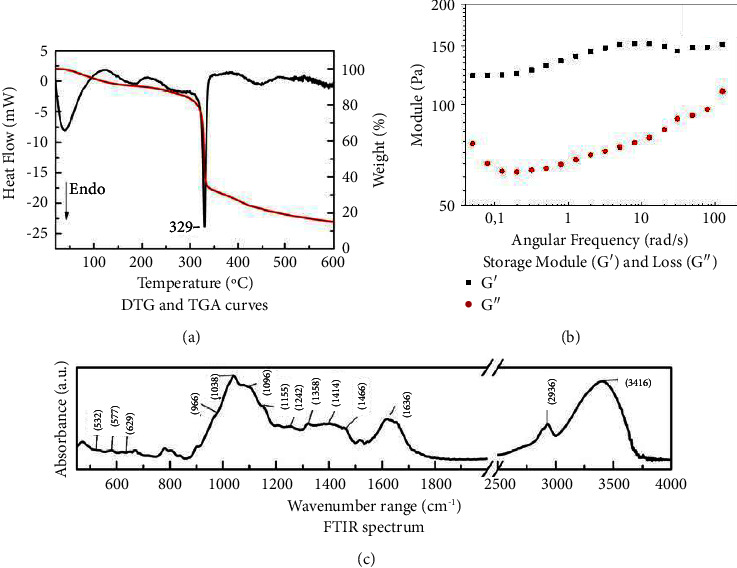
Characterization of “canela-guaicá” bark extract (CGBE) by thermogravimetric analysis (TGA) ((a), redline), derivative thermogravimetric analysis (DTG) ((a), blackline), rheological analysis (b), and fourier-transform infrared (FTIR) spectrum (c).

Figure 1(a) depicts the thermal results of TGA and DTG. TGA and DTG curves showed four events of mass loss. The thermal gravimetric data of the CGBE sample recorded a decomposition process starting at 38°C. This event may be due to the thermal desorption of the remaining free water molecules in the sample, causing a small amount of mass loss with a loss rate of 4.4%. A second event of water loss occurred at 174°C and was assigned to the water adsorbed to carbohydrate polymers due to the binding of functional groups. After a period of heating up, the temperature reached 329°C and entered the third stage of decomposition. The sample revealed a large amount of mass loss with a loss rate of 53.8% related to the alkaloid and mucilage compounds. The temperature continued to rise and the sample further decomposed at 445°C, and the remaining sample mass was 19.3%. This event was attributed to amino group degradation. The obtained data by TGA/DTG revealed a regular mass degradation, which indicated that CGBE showed high purity and could be used as a phytotherapeutic material for wound healing. Furthermore, the thermal degradation of the CGBE compounds occurred above 300°C, which makes it suitable for the proposed use as a dressing for wounds, since the temperature of human skin is remarkably lower.

Considering the rheological data, the storage modulus (*G*′) and the loss modulus (*G*″) versus angular frequency (rad/s) plots for the CGBE sample are described in Figure 1(b). CGBE presented a higher elastic modulus (*G*′) than viscous modulus (*G*″), and there was no crossing point between *G*′ and *G*″′ for the range of angular frequencies applied. In that sense, CGBE demonstrated a pseudoplastic behavior that enabled it to be deformable as the share force increased. This material showed resistance to mechanical stresses such as tearing, shearing, and elongation, which is an important feature for healing applications [27, 28].

The FTIR spectrum of CGBE is summarized in Figure 1(c). A broad O-H band at 3,416 cm^−1^ was assigned to the mucilage polysaccharides. The band at 2,936 cm^−1^ was attributed to the alkyl C-H stretching. The aporphine alkaloids presented bands at 1,466 and 1,414 cm^−1^ due to the aromatic ring. The FTIR data confirmed the results obtained in the phytochemical qualitative analysis and demonstrated the potential of CGBE for wound [24] and pain [12] treatment since mucilage and alkaloids have been reported as healing compounds [29] and angiogenic and anti-inflammatory substances [30], respectively. However, these findings lack in vivo biological evidence to validate the CGBE traditional use by indigenous Kaingang groups in southern Brazil.

### 3.2. Preclinical Trial: Wound Healing Assay

**Figure 2:**
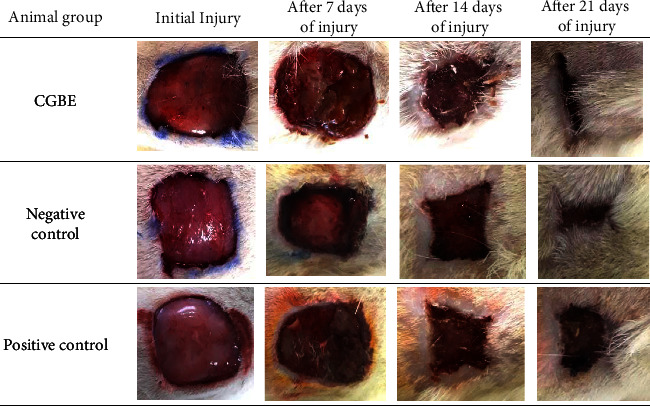
Macroscopic aspect of wounds for experimental groups at different time intervals. Images are representative of wounds in Wistar rats using a standard photography apparatus. Note: CGBE – “canela-guaicá” bark extract.

The results of the macroscopic evaluation for experimental groups are illustrated in Figure 2. The CGBE group showed an increased lesion area after seven days of injury as well as the positive control. These findings are expected in the inflammatory phase of the murine lesion since the inflammation proceeds as usual and the dominant macrophage population shifts from an inflammatory to a tissue repair phenotype characterized by secretion of various paracrine factors. These factors promote angiogenesis as well as further proliferation of fibroblastic cells, mesenchymal stem/stromal cells, and other local stem and progenitor cells [31, 32].

After 14 days of injury, the proliferative phase of wound healing is typically occurring, and the new connective tissue or granulation tissue is formed concurrently with other healing processes, including re-epithelialization, neovascularization, and immune modulation [33]. However, the macroscopic images of the lesions do not allow to examine these processes, and further histological data are required to confirm the healing progression.

After 21 days of injury, CGBE demonstrated a substantial healing potential with a suitable regression of murine skin lesions. This performance was distinctly superior to the positive control, as shown in Figure 2. In particular, the negative control presented the lesion regression behavior in an intermediate degree between the CGBE group and the positive control due to the natural course of the healing process, as previously reported [34, 35].

These data can also be demonstrated by the wound healing index (WHI) as represented in Table 1. The WHI values were consistent with the observational data provided by the macroscopic evaluation. In particular, CGBE was the only experimental group that provided lesion regressions of above 90%, which denotes its positive effect on wound healing.

**Figure 3:**
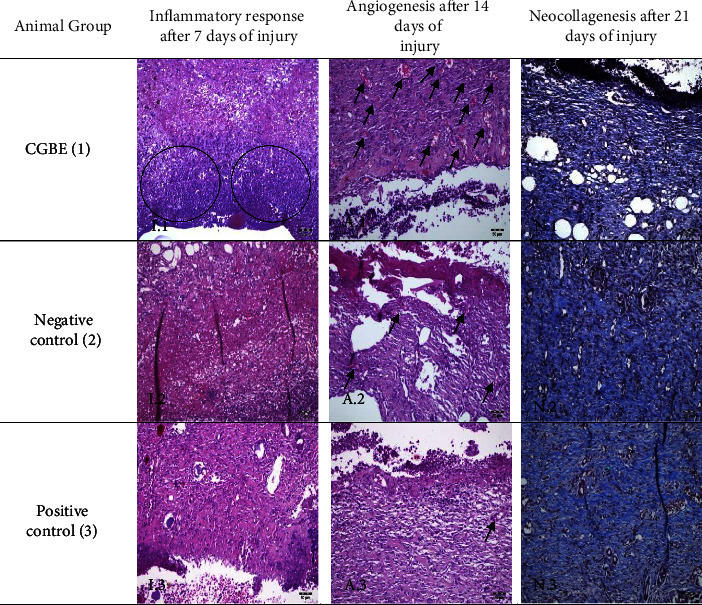
Microscopic aspect of wounds throughout the experiment according to such criteria: inflammatory response, angiogenesis, and neocollagenesis for experimental groups at different time intervals. Circles areas reveals a increased presence of inflammatory cells (poly and mononuclear). Arrows indicate new vessels. Several microphotographs were taken using a × 200 magnification and the images are representative of the experimental results. Note: CGBE – “canela-guaicá” bark extract.

Considering the promising macroscopic and WHI findings, histological analysis was carried out in order to elucidate some probable processes involved in the wound healing progression such as the inflammatory response, the angiogenesis, and the neocollagenesis after 7, 14, and 21 days of injury, respectively. These results are described in Figure 3 and Table 2.

CGBE demonstrated a statistically higher histological score than the respective controls for the inflammatory response. In addition, an intense concentration of poly and mononuclear inflammatory cells (purple nuclei) was observed in the extracellular matrix and inflammatory infiltrate in the CGBE-treated group after 7 days, when compared to the other groups (Figure 3, photomicrograph I.1). This enhanced inflammatory process, but not excessive, was essential for the inflammatory phase of wound healing since it allowed a suitable activation of cellular and humoral elements fundamental to healing. In addition, the process of wound inflammation is crucial for the optimal completion of hemostasis, as well as the detection and elimination of pathogenic microorganisms, the removal of damaged tissues, and wound cleaning. Furthermore, leukocytes (mainly macrophages) are responsible for the timely and adequate preparation of wound tissues for proliferation [36, 37].

Significant angiogenesis was reported for the CGBE group after 14 days of injury when compared to control groups. The formation of new vessels (arrows) is observed in higher concentration in the CGBE group when compared to the other groups (Figure 3, photomicrograph A.1). Remarkably, these findings could not be verified by the macroscopic analysis and were only histologically observed. This experimental evidence may be related to the presence of aporphine alkaloids in the CGBE composition. Alkaloids, according to previous studies, can stimulate new vessels in lesions and can promote fibroblasts proliferation and/or collagen production [24–26, 29, 38]. As a result of this neovascularization, with suitable supply of micronutrients and tissue oxygenation [32], a better lesion regression was macroscopically achieved after 21 days of injury.

Considering the neocollagenesis, no statistically significant difference was obtained. The acute wound in a healthy rat model has a rapid course and is usually closured in 21 days, which provides an expanded window of observation and identification of critical events involved in wound repair [39]. Thus, the end of the acute process for all groups did not allow for establishing differences between them.

The results highlighted in this paper revealed the promising healing effect of CGBE; however, a study limitation must be reported. Some additional experiments such as enzyme-linked immunosorbent assay (ELISA), western blotting or immunohistochemistry for inflammatory cytokines and vascular endothelial growth factor (VEGF) expression, and alpha-smooth muscle actin (*α*-SMA) for angiogenesis could be further performed in order to elucidate the possible mechanism of action.

In brief, CGBE was essential in the inflammatory and proliferative stages of wound healing due to the improvement of cell migration and angiogenesis [40–42]. In that sense, the healing potential of CGBE, traditionally used by the Kaingang indigenous community from southern Brazil, was preclinically confirmed. This study can support the design of novel wound dressings containing this ethnopharmacological-based strategy for wound repair.

## 4. Conclusion

The mucilaginous “canela-guaicá” bark extract (CGBE) was effective as an indigenous traditional practice for treating wounds. CGBE presented alkaloids and polysaccharides as the main chemical constituents. This natural product showed thermal stability and pseudoplastic rheological behavior. CGBE provided a remarkable regression of skin lesions in the macroscopic evaluation of tissue repair after 21 days. The histological analysis demonstrated an enhanced inflammatory response and angiogenesis. CGBE proved to be an expressive ethnopharmacological knowledge of the Kaingang community from southern Brazil.

## Figures and Tables

**Table 1:** Wound healing index (WHI) for the experimental groups at different time intervals.

Animal group	WHI (%)		
	After 7 days of injury	After 14 days of injury	After 21 days of injury
	Mean ± SD	Mean ± SD	Mean ± SD
CGBE	−7.8 ± 1.7^a^	+64.1 ± 3.2^a^	+95.4 ± 3.3^a^
Negative control	+1.8 ± 1.1^b^	+63.6 ± 4.7^a^	+87.8 ± 5.9^a,b^
Positive control	−5.5 ± 2.2^a^	+68.9 ± 4.5^a^	+78.8 ± 4.9^b^

^*∗*^one-way nonparametric ANOVA (Kruskal–Wallis test); different letters on the columns indicate the statistical difference (*p* < 0.05). *Note*. CGBE, “canela-guaicá” bark extract; SD, standard deviation.

**Table 2:** Histological scores of wounds throughout the experiment according to such criteria: inflammatory response, angiogenesis, and neocollagenesis for experimental groups at different time intervals.

Animal group	Inflammatory response after 7 days of injury	Angiogenesis after 14 days of injury	Neocollagenesis after 21 days of injury
	Mean ± SD	Mean ± SD	Mean ± SD
CGBE	2.20 ± 0.33^a^	2.40 ± 0.06^a^	2.00 ± 0.71^a^
Negative control	1.00 ± 0.00^b^	1.60 ± 0.33^b^	2.40 ± 0.55^a^
Positive control	1.00 ± 0.00^b^	1.20 ± 0.25^b^	2.60 ± 0.55^a^

^*∗*^one-way nonparametric ANOVA (Kruskal–Wallis test); different letters on the columns indicate the statistical difference (*p* < 0.05). *Note*. CGBE, “canela-guaicá” bark extract; SD, standard deviation.

## Data Availability

The data used to support the findings of the study are available from the corresponding author upon request.

## Additional Points

“Canela-guaicá” [*Ocotea puberula* (Rich.) Nees] bark extract (CGBE) presented mucilage and alkaloids. This natural product showed thermal stability and pseudoplastic properties. A statistically higher angiogenesis was observed for CGBE after 14 days of treatment. An improved macroscopic wound repair was observed for CGBE after 21 days.
